# Occurrence and genotyping of *Giardia duodenalis* and *Cryptosporidium* in pre-weaned dairy calves in central Sichuan province, China

**DOI:** 10.1051/parasite/2018046

**Published:** 2018-09-04

**Authors:** Zhijun Zhong, Jiaming Dan, Guangwen Yan, Rui Tu, Yinan Tian, Suizhong Cao, Liuhong Shen, Junliang Deng, Shumin Yu, Yi Geng, Xiaobin Gu, Ya Wang, Haifeng Liu, Guangneng Peng

**Affiliations:** 1 Key Laboratory of Animal Disease and Human Health of Sichuan Province, College of Veterinary Medicine, Sichuan Agricultural University Sichuan 611130 PR China; 2 College of Animal Science, Xichang University Xichang 615000 PR China

**Keywords:** *Giardia duodenalis*, *Cryptosporidium*, Multilocus genotyping, Pre-weaned dairy calves, Sichuan province

## Abstract

*Giardia duodenalis* and *Cryptosporidium* spp. are common human and animal pathogens. They have increasingly been reported in dairy calves in recent years; however, multilocus genotyping information for *G. duodenalis* and *Cryptosporidium* infecting pre-weaned dairy calves in southwestern China is limited. In the present study, the prevalence of *G. duodenalis* and *Cryptosporidium* spp. in pre-weaned dairy calves in central Sichuan province was determined and the pathogens were analyzed molecularly. Of 278 fecal samples from pre-weaned dairy calves, 26 (9.4%) were positive for *G. duodenalis* and 40 (14.4%) were positive for *Cryptosporidium* spp. *Cryptosporidium bovis* (*n* = 28), *Cryptosporidium ryanae* (*n* = 5) and *Cryptosporidium parvum* (*n* = 7) were detected. All seven *C. parvum* isolates were successfully subtyped based on the *gp60* gene sequence, and only IIdA15G1 was detected. Multilocus sequence typing of *G. duodenalis* based on beta-giardin (*bg*), triose phosphate isomerase (*tpi*) and glutamate dehydrogenase (*gdh*) genes revealed 19 different assemblage E multilocus genotypes (two known and 17 unpublished genotypes). Based on eBURST analysis, a high degree of genetic diversity within assemblage E was observed in pre-weaned dairy calves in Sichuan province. To the best of our knowledge, this is the first study using multilocus sequence typing and eBURST analysis to characterize *G. duodenalis* in pre-weaned dairy calves in southwestern China.

## Abstract (fr)

*Giardia duodenalis* et *Cryptosporidium* spp. sont des pathogènes humains et animaux communs. Ils ont été signalés de plus en plus chez les veaux laitiers au cours des dernières années; cependant, l’information de génotypage multilocus pour *G. duodenalis* et *Cryptosporidium* infectant les veaux laitiers pré-sevrés dans le sud-ouest de la Chine est limitée. Dans la présente étude, la prévalence de *G. duodenalis* et de *Cryptosporidium* spp. chez les veaux laitiers pré-sevrés dans la province centrale du Sichuan a été déterminée et les pathogènes ont été analysés moléculairement. Dans 278 échantillons fécaux de veaux laitiers pré-sevrés, 26 (9.4 %) étaient positifs pour *G. duodenalis* et 40 (14.4 %) étaient positifs pour *Cryptosporidium* spp. *Cryptosporidium bovis* (*n* = 28), *Cryptosporidium ryanae* (*n* = 5) et *Cryptosporidium parvum* (*n* = 7) ont été détectés. Les sept isolats de *C. parvum* ont été sous-typés avec succès sur la base de la séquence du gène gp60 et seul IIdA15G1 a été détecté. Le typage multilocus de *G. duodenalis* basé sur les gènes de béta-giardine (bg), triose phosphate isomérase (tpi) et glutamate déshydrogénase (gdh) a révélé 19 génotypes différents d’assemblage multilocus E (deux génotypes connus et 17 non-publiés). D’après l’analyse eBURST, on a observé un degré élevé de diversité génétique au sein de l’assemblage E chez les veaux laitiers pré-sevrés de la province du Sichuan. À notre connaissance, il s’agit de la première étude utilisant le typage de séquence multilocus et l’analyse eBURST pour caractériser *G. duodenalis* chez des veaux laitiers pré-sevrés dans le sud-ouest de la Chine.

## Introduction

Protists of the genera *Giardia* and *Cryptosporidium* infect a wide range of animals as well as humans [3, 12, 19]. Typically, the infection is acquired following the ingestion of highly resilient, infective stages (oocysts or cysts) via the fecal-oral route [3, 4]. Disease is commonly associated with clinical signs including diarrhea, dehydration, fever, inappetence and anorexia. Infections are often self-limiting in immune-competent individuals [2, 31], but can be chronic and severe in infants, elderly people, and immune-compromised individuals [9, 16].

Ruminants are recognized as a significant reservoir of *Giardia* and *Cryptosporidium* taxa that infect animals and humans [19, 21]. Current data indicate that of the eight assemblages within *Giardia duodenalis*, assemblages A and E and the *Cryptosporidium* species *C. parvum, C. andersoni, C. ryanae,* and *C. bovis* predominate in cattle worldwide [4, 20].

Unlike in other countries (e.g. Australia, Sudan, Japan and India) [6, 14, 15, 24], where *C. parvum* is known to be the predominant species in pre-weaned calves, this does not appear to be the case everywhere in China. Some studies have shown that *C. parvum* is a major species in pre-weaned calves in some regions, whereas *C. bovis* is a major species in other regions [5, 25, 28].

According to the National Bureau of Statistics of the People’s Republic of China, in 2016, the total population of dairy cattle in Sichuan Province was 176 thousand heads. However, no information about *G. duodenalis* and *Cryptosporidium* infection of pre-weaned dairy calves was previously available in Sichuan Province. We undertook a molecular epidemiological study to obtain a preliminary snap-shot of the prevalence of *G. duodenalis* assemblages and *Cryptosporidium* genotypes in pre-weaned calves in Sichuan province, China.

## Materials and methods

### Sample collection

**Figure 1.**
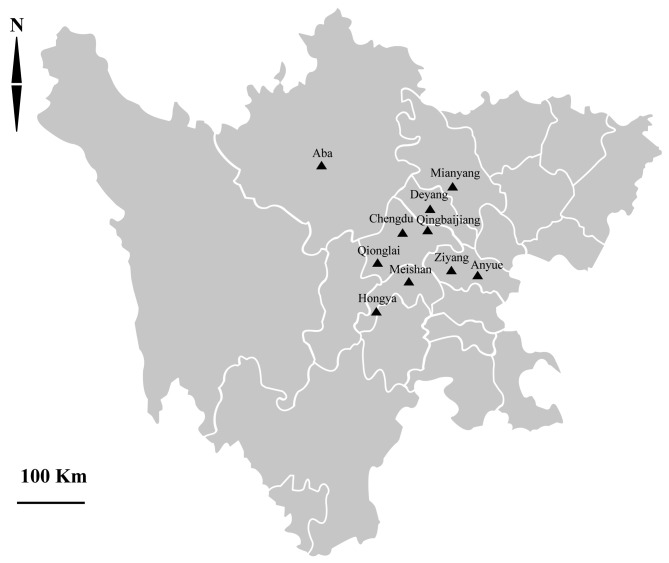
Distribution of sampling sites in Sichuan province in this study.

We collected 278 rectal fecal samples from pre-weaned dairy calves (<1 month of age) from 10 farms with a history of bovine diarrhea in 10 regions in Sichuan province, southwestern China, between June 2016 and March 2017. Collection sites included: Chengdu (104°06′E, 30°57′N), Hongya (103°37′E, 29°91′N), Aba (102°22′E, 31°90′N), Meishan (103°84′E, 30°08′N), Mianyang (104°67′E, 31°47′N), Ziyang (104°62′E, 30°13′N), Anyue (105°33′E, 30°10′N), Qionglai (103°46′E, 30°41′N), Qingbaijiang (104°25′E, 30°88′N), and Deyang (104°39′E, 31°13′N). The 10 farms are distributed in central Sichuan Province (Fig. 1). The city-level map was provided by the National Geomatics Centre of China (National Geomatics Centre of China, Beijing, China, http://ngcc.sbsm.gov.cn/).

Of the 10 farms, six (Chengdu, Hongya, Aba, Mianyang, Ziyang, and Qionglai) are intensive feeding farms, while the other four are free-ranging. For intensive farms, there were approximately 1000–2500 cattle per farm, with more than 100 pre-weaned dairy calves, and the fecal samples were randomly collected from about 20% in each farm. For free-ranging farms, there were approximately 100–120 cattle per farm and the herd sizes of pre-weaned dairy calves were less than 50; in this case we collected fecal samples from all of the pre-weaned dairy calves at each farm (Table S1). In intensive farms, calves were bred in different calf stalls, with one hour outdoor time after eating and excretion in the morning and afternoon, respectively. Calves shared one yard during the outdoor time in intensive farms. In free-ranging farms, calves were kept in a field with a half cover and were raised together. The farms we selected had solely cattle, and no other animals.

Fecal samples were collected from the rectum using disposable gloves, transferred into disposable plastic bags, and stored in 2.5% potassium dichromate at 4 °C.

### DNA extraction

Before DNA extraction, feces were washed with distilled water to remove potassium dichromate. Genomic DNA was extracted from 250 mg (approximately) of individual samples using the Power Soil DNA isolation kit (MOBIO, USA), according to the manufacturer’s instructions, and frozen at −20 °C until use.

### PCR amplification and sequencing

*G. duodenalis* was detected by nested PCR amplification of the *bg*gene. The *bg*-positive samples were further characterized by amplifications of *gdh* and *tpi*. Genotyping of *Cryptosporidium* was based on amplification of the small subunit (SSU) rRNA gene by nested PCR and subsequent sequence analysis. All the *C. parvum* isolates were further characterized by amplification of the *gp60* gene. The primers and amplification conditions in this study were described previously [1, 11, 23]. Positive and negative controls were included in each test. The secondary PCR products were visualized under UV light after electrophoresis on a 1% agarose gel mixed with Golden View.

All positive secondary PCR products were sent to Invitrogen (Shanghai, China) and sequenced in both directions. Sequences were aligned with reference sequences from GenBank using BLAST (http://blast.ncbi.nlm.nih.gov) and ClustalX.

A previous nomenclature system was used to name subtypes at each genetic locus [29, 30]. Specimens that were successfully subtyped at all three loci were included in multilocus genotyping of *G. duodenalis*. The genetic pedigree of the assemblage E multilocus genotypes (MLGs) was assessed by using eBURST 3.0 (http://eBURST.mlst.net).

### Statistical analysis

The *χ*^2^ test was used to compare the infection rates of *G. duodenalis* and *Cryptosporidium* in different feeding patterns. Differences were considered significant at *p* < 0.05.

## Results and discussion

*G. duodenalis* was detected in 9.4% of 278 pre-weaned dairy calves on 6 of 10 farms, with prevalences ranging from 7.7% to 46.4% (Table 1). Its prevalence shows substantial differences, ranging from 7.1% to 60.1% in other studies in China [7, 13, 18, 26, 29]. In this study, the overall infection rate in southwestern China was close to the prevalence in northwestern (9.7% [18]), northeastern (13.3% [13]) and north China (7.1% [7]), but much lower than the infection rates in central (17.6% [26]) and southeastern (60.1% [29]) China. Prior to the present study, these results were interpreted as related to differences in geographic distribution, environmental management and cultivation scale [7, 13, 18, 26, 29]. Cattle were kept in groups or in free stalls, which might promote the transmission of *G. duodenalis* infection among animals and lead to the high infection rates [26, 29]. Furthermore, we analyzed the infection rates between intensive feeding and free-ranging farms; there was no significant difference between the two breeding patterns (*X* ^2^ = 0.629, *df* = 1, *p* = 0.428).

**Table 1.** Prevalence of *Cryptosporidium* and *G. duodenalis* in pre-weaned diary calves in Sichuan province.

Region	No. tested	*Cryptosporidium*			*Cryptosporidium* No. (%) of positive specimens	*G. duodenalis*	*G. duodenalis* infection rate
		*C. bovis*	*C. ryanae*	*C. parvum*			
Chengdu^a^	39	2	1		3 (7.7%)	3	7.7%
Hongya^a^	24	1			1 (4.2%)		
Aba^a^	20			7	7 (35.0%)	2	10.0%
Meishan^b^	20						
Mianyang^a^	58	8	3		11 (19.0%)		
Ziyang^a^	26	8			8 (30.8%)	2	7.7%
Anyue^b^	22	2			2 (9.1%)	3	13.6%
Qionglai^a^	28	4			4 (14.3%)	13	46.4%
Qingbaijiang^b^	20	2			2 (10.0%)	3	15.0%
Deyang^b^	21	1	1		2 (9.5%)		
Total	278	28	5	7	40 (14.4%)	26	9.4%

a Intensive farming;

b free-ranging.

*Cryptosporidium* was detected in 14.4% of 278 fecal samples, on 9 out of 10 farms, with prevalences ranging from 4.2% to 35.0% (Table 1). The overall infection rate for *Cryptosporidium* is lower than the average prevalence of 19.5% reported previously in pre-weaned cattle in China [5], but similar to the infection rate reported in Xinjiang (15.6%) [17], and much higher than the rate in Hebei and Tianjin, China (1.0%) [7]. Prevalence of *Cryptosporidium* was significantly different (*X* ^2^ = 4.924, *df* = 1, *p* = 0.026) between intensive feeding and free-ranging farms in this study, which suggests that cultivation scale may lead to differences in infection rates with *Cryptosporidium*. Other studies also showed that geographic distribution and host health status may lead to the difference [5, 10, 17]. Three species of *Cryptosporidium* (28 *C. bovis*, 7 *C. parvum* [subtype IIdA15G1] and 5 *C. ryanae*) were identified in this study. Previous studies have shown that *C. parvum* is a major species in pre-weaned calves in Beijing [10], Xinjiang [17] and Ningxia [8], whereas *C. bovis* predominated in pre-weaned calves in this study, similar to reports from Henan [27] and Heilongjiang [32].

*Giardia duodenalis* in all 26 positive samples corresponded to assemblage E. *G. duodenalis* infection is relatively common in pre-weaned dairy calves. We further characterized the 26 *G. duodenalis bg*-positive samples at the *tpi* and *gdh* loci. Among these 26 samples, the *tpi* and *gdh* loci were successfully amplified and sequenced in 24 and 25 specimens, respectively. The *bg*, *tpi* and *gdh* loci all showed high levels of sequence polymorphism; seven subtypes were identified at each locus. Of the *bg* subtypes, E1 (MF671885), E8 (KY769093), E9 (KY769091), and E15 (KT698677) were known, and E13 (MF671880), E14 (MF671883), and E16 (MF671886) were unpublished. At the *tpi* locus, five known subtypes E1 (MF671900), E3 (KT922259), E9 (EF654690), E15 (KY432848) and E19 (KY769103) and two unpublished subtypes, E21 (MF671904) and E24 (MF671907), were found. The sequences from the *gdh* locus represented five known subtypes E1 (MF671891), E3 (KT369780), E8 (KT368785), E10 (KT698971), E13 (KY432838) and two unpublished subtypes, E19 (MF671896) and E20 (MF671899).

**Figure 2.**
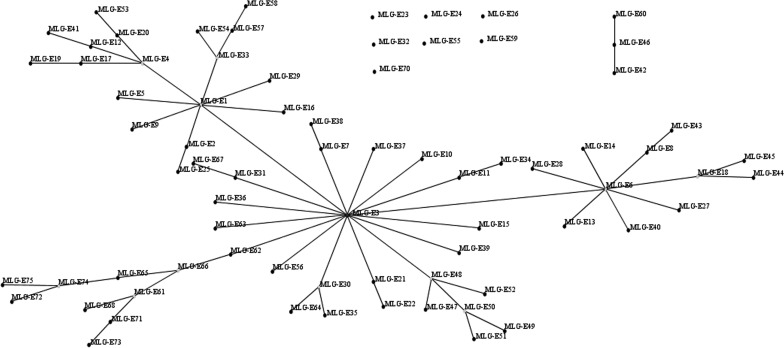
eBURST networks for *G. duodenalis* assemblage E. Each MLG is represented by a dot. MLG-E3 is the primary founder, and the subgroup founders are MLG-E1, MLG-E4, MLG-E33, MLG-E66, MLG-E61, MLG-E74, MLG-E30, MLG-E48, MLG-E50, MLG-E6, and MLG-E18. The variants are connected by lines.

For *G. duodenalis*, multi-locus genotyping analysis suggested a high genetic diversity of assemblage E in pre-weaned dairy calves in this study. Based on the combination of *bg*, *tpi* and *gdh* loci, 19 MLGs of assemblage E were detected (Table 2). A high degree of nucleotide variation in assemblage E has been also detected in previous studies [18, 22, 26, 29]. Of the 19 MLGs, 17 were unpublished MLGs. The majority of MLGs were MLG-E3 and MLG-E13, which have also been detected in dairy calves in Shanghai [29]. To further analyze the evolutionary descent of the 19 assemblage E MLGs, we used eBURST analysis of the 19 assemblage E MLGs and 58 reference MLGs, which revealed two clonal complexes and seven singletons (Fig. 2). MLG-E3 is the primary founder of clonal complex 1, which is consistent with findings in a previous study in Shanghai [29]. The majority of MLGs (14/19) originated from MLG-E3. Furthermore, MLG-E60 is a variant of clonal complex 2, and MLG-E59 and MLG-E70 were singletons. The latter three MLG subtypes showed distant evolution from other assemblage E MLGs, which may indicate substantial differences in their evolutionary divergence [29].

**Table 2.** Multilocus sequence genotypes of *G. duodenalis* in pre-weaned dairy calves in Sichuan province.

Isolate	Geographic source	Subtype			MLG
		*bg*	*tpi*	*gdh*	
ABG3417	A’ba	E9	E15	#E19/MF671896	#MLGE72
ABG3422		E1	E15	#E19/MF671896	#MLG E74
AYG6943	Anyue	#E13/MF671880	E1	E10	#MLG E70
AYG6950		#E14/MF671883	E3	E10	#MLG E67
AYG6953		E1	E3	E3	#MLG E62
CDG16089	Chengdu	E9	E3	E1	#MLG E61
CDG16090		E8	E9	E10	#MLG E60
CDG16100		E9	E19	E1	#MLG E68
QBJG13	Qingbaijiang	#E14/MF671883	E3	E10	#MLG E67
QBJG17		#E16MF671886	E3	E3	#MLG E63
QBJG18		E9	E3	E10	MLG E3
QLG5065	Qionglai	E1	#E24MF671907	#E19/MF671896	#MLG E75
QLG5066		E9	E3	E10	MLG E3
QLG5067		E1	E3	E8	#MLG E64
QLG5070		E1	E15	E1	#MLG E65
QLG5071		E9	E1	#E19MF671896	#MLG E73
QLG5073		E1		E10	
QLG5074		E1	E15	E1	#MLG E65
QLG5075		#E13/MF671880	E3	E1	MLG E13
QLG5076		#E13/MF671880	E3	#E20/MF671899	#MLG E59
QLG5083		E1	E3	E1	#MLG E66
QLG5091		#E13/MF671880	E3	E1	MLG E13
QLG5092		#E13/MF671880	E3	E1	MLG E13
QLG5093		#E13			
ZYG6863	Ziyang	E9	E1	E1	#MLGE71
ZYG6844		E15	#E21/MF671904	E13	#MLG E69

# Unpublished subtypes and MLGs.

## Conclusion

This is the first study to genotype *G. duodenalis* and *Cryptosporidium* in pre-weaned dairy calves in Sichuan province. *C. bovis* and *G. duodenalis* assemblage E are the dominant species in pre-weaned dairy calves in Sichuan, and high genetic diversity of assemblage E MLGs was observed.

## Supplementary Material

Table S1 is available at https://www.parasite-journal.org/10.1051/parasite/2018023/olm

## Statements

### Availability of data and material

The datasets supporting the conclusions of this article are included within the article. *Cryptosporidium* and *G. duodenalis* sequences were deposited in the GenBank database under accession numbers MF671870–MF671908.
