# Supplementary material for: Occurrence and genotyping of Giardia duodenalis and Cryptosporidium in pre-weaned dairy calves in central Sichuan province, China
Source: Parasite. 2018 Sep 4;25:45. doi: 10.1051/parasite/2018046 (PMC6121785; doi:10.1051/parasite/2018046)
Supplement: Table S1 is available at https://www.parasite-journal.org/10.1051/parasite/2018023/olm [file parasite-25-45-s1.docx]

Table S1 Number of pre-weaned dairy calves and number of sampled in each farm.

| Regions | Number of pre-weaned dairy calves (<1 month) in each farm | Number of sampled |
| --- | --- | --- |
| Chengdu^a^ | 198 | 39 |
| Hongya^a^ | 123 | 24 |
| Aba^a^ | 104 | 20 |
| Meishan^b^ | 20 | 20 |
| Mianyang^a^ | 291 | 58 |
| Ziyang^a^ | 132 | 26 |
| Anyue^b^ | 22 | 22 |
| Qionglai^a^ | 144 | 28 |
| Qingbaijiang^b^ | 20 | 20 |
| Deyang^b^ | 21 | 21 |
| Total | 1075 | 278 |

^a^ : intensive farming

^b^ : free-ranging
